# Understanding the Role of Protein Glycation in the Amyloid Aggregation Process

**DOI:** 10.3390/ijms22126609

**Published:** 2021-06-21

**Authors:** Ivana Sirangelo, Clara Iannuzzi

**Affiliations:** Department of Precision Medicine, Università degli Studi della Campania “Luigi Vanvitelli”, Via L. De Crecchio 7, 80138 Naples, Italy; ivana.sirangelo@unicampania.it

**Keywords:** amyloid aggregation, protein glycation, AGEs, protein misfolding, amyloidosis

## Abstract

Protein function and flexibility is directly related to the native distribution of its structural elements and any alteration in protein architecture leads to several abnormalities and accumulation of misfolded proteins. This phenomenon is associated with a range of increasingly common human disorders, including Alzheimer and Parkinson diseases, type II diabetes, and a number of systemic amyloidosis characterized by the accumulation of amyloid aggregates both in the extracellular space of tissues and as intracellular deposits. Post-translational modifications are known to have an active role in the in vivo amyloid aggregation as able to affect protein structure and dynamics. Among them, a key role seems to be played by non-enzymatic glycation, the most unwanted irreversible modification of the protein structure, which strongly affects long-living proteins throughout the body. This study provided an overview of the molecular effects induced by glycation on the amyloid aggregation process of several protein models associated with misfolding diseases. In particular, we analyzed the role of glycation on protein folding, kinetics of amyloid formation, and amyloid cytotoxicity in order to shed light on the role of this post-translational modification in the in vivo amyloid aggregation process.

## 1. Introduction

The increase in life expectancy observed over the last century has led to the appearance of a new set of pathologies that constitute new challenges to scientists and clinicians. Among these, neurodegenerative disorders like Alzheimer’s, Parkinson’s, and prion diseases are debilitating and incurable disorders with an increasing impact on society, because the number of diagnosed patients has dramatically increased over the past twenty years and it is expected to further increase in developing countries [1,2]. The histological hallmark of these disease is the presence of proteinaceous aggregates, which form deposits called amyloid plaques that usually accumulate both in the extracellular space of tissues and as intracellular deposits [3,4]. Although the amyloid aggregation process of the proteins involved has been widely characterized in vitro, the molecular mechanisms underlying the formation of amyloid species in vivo and in pathological conditions are still poorly understood. In this respect, post-translational modifications are known to have an active role as able to affect protein structure and dynamics [5,6]. Among them, a key role seems to be played by non-enzymatic glycation, an irreversible modification of the protein structure, which strongly affects long-living proteins throughout the body [7,8]. Indeed, proteins in amyloid deposits are often found glycated in patients, thus suggesting a direct correlation between protein glycation and amyloidosis [9,10,11,12]. For this reason, much attention has been paid to the role played by non-enzymatic glycation in promoting amyloid aggregation and cytotoxicity. This study provided an overview of the molecular effects induced by glycation on the amyloid aggregation process of several protein models associated with misfolding diseases in order to shed light on the role of this post-translational modification in the in vivo amyloid formation.

## 2. Protein Glycation

During their lifetime, proteins are exposed to several altering factors, including enzymatic and non-enzymatic mechanisms. Among the non-enzymatic mechanisms, protein glycation is one of the most important post-translational modification in which protein is covalently modified through the addition of functional groups to its amino-acid residues [13,14]. This process is different from glycosylation which is a selective protein modification driven by specific enzymes, generally associated with a gain of function (or stabilization) of the target protein. Differently, non-enzymatic glycation is a non-selective modification and it is generally associated with a loss of function of the target protein due to modifications of its native structure.

**Figure 1:**
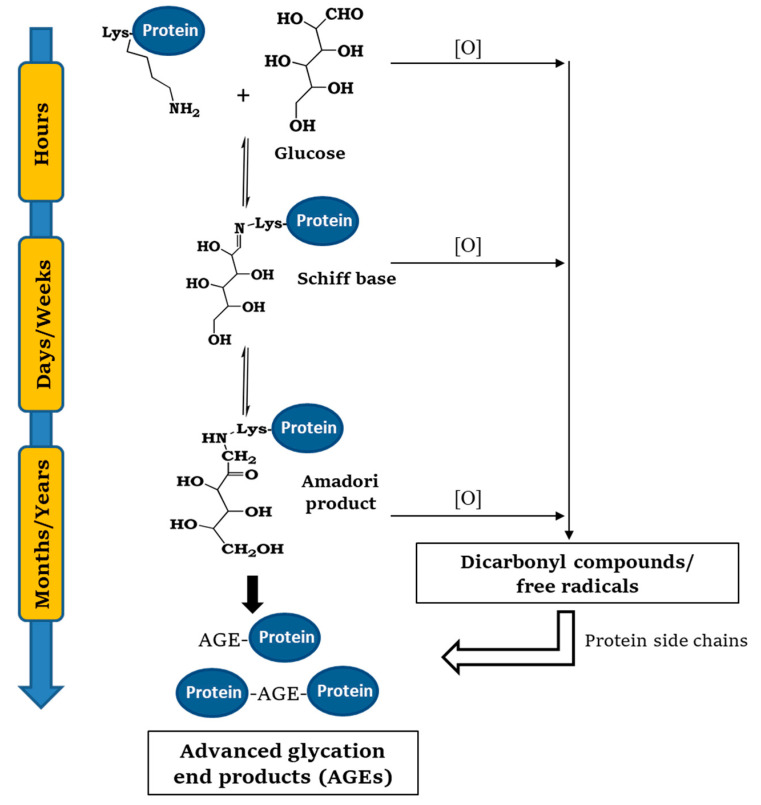
General mechanism of protein glycation. The process is initiated by a spontaneous nucleophilic addition reaction between the free amino group of a protein, generally belonging to N-terminal and lysine side chain, and the carbonyl group of a reducing sugar. This reaction rapidly forms a reversible Schiff base, which rearranges over a period of weeks to produce ketoamine or Amadori product. The Amadori product undergoes an irreversible cascade of reactions involving dehydration, hydrolysis, and rearrangements leading to the formation of advanced glycation end products (AGEs).

Glycation reaction is a naturally occurring process common to all cell types: Glycated products slowly accumulate in vivo leading, in addition to cellular modifications involved in the aging process, to several different protein dysfunctions [8,15,16]. Protein glycation is initiated by a spontaneous nucleophilic addition reaction between the free amino group of a protein, generally belonging to N-terminal and lysine side chain, and the carbonyl group of a reducing sugar. This reaction rapidly forms a reversible Schiff base, which rearranges over a period of weeks to produce ketoamine or Amadori product. This reaction is reversible depending on the concentration of the reactants. Thereafter, the Amadori product undergoes an irreversible cascade of reactions involving dehydration, hydrolysis, and rearrangements leading to the formation of advanced glycation end products (AGEs) (Figure 1) [17,18].

Although the formation of the Schiff base and the Amadori product constitutes the central pathways along this mechanism, the whole process becomes much more complex due to collateral autoxidative reactions of reducing sugars, Schiff bases, and the Amadori product. In particular, these reactions produce highly reactive carbonyl species and free radicals that can further react with free amino acid side chains contributing to the AGEs formation [19,20] (Figure 1). In fact, although glycation can be started by all reducing sugars, the activity of dicarbonyl compounds like glyoxal (GO) and methylglyoxal (MGO) in the reaction is much higher even at negligible concentrations [20]. The main targets of protein glycation are side-chains of arginine and lysine residues, the N-terminus amino group, and thiol groups of cysteine residues. The kinetics of the process depends on several conditions: concentration and reactivity of the glycation agent, the presence of catalytic factors (metals, buffer ions, and oxygen), pH, temperature, exposure of glycating sites, and half-life of the protein. All reducing sugars can promote glycation reactions and, between them, D-ribose is the most active, while D-glucose, glucose 6-phosphate, mannose, and fructose are much less reactive [16,21].

**Figure 2:**
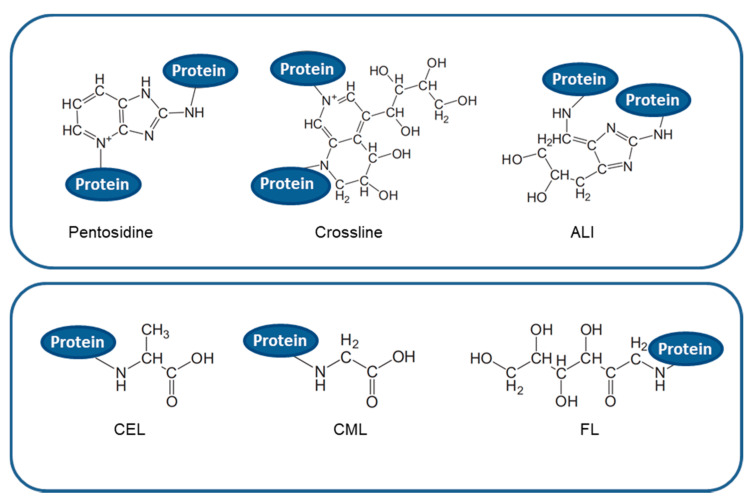
Chemical structure of different type of AGEs. Upper panel: cross-linking AGEs such as pentosidine; crossline; and ALI: arginine-lysine imidazole cross-links. Lower panel: non-cross-linking AGEs such as CEL: N-carboxyethyl-lysine; CML: N-carboxymethyllysine; and FL: N-fructosyl-lysine.

Glycated species are very heterogeneous and generally classified in cross-linking and non-cross-linking AGEs on the basis of their ability to form covalent cross-links, both intra- and inter-molecular, within the polypeptide chains (Figure 2). AGEs accumulate slowly throughout lifetime and are considered a marker for several diseases, such as arteriosclerosis, renal failure, Alzheimer disease, or diabetes, although they normally increase in aging [22]. In fact, due to their chemical, pro-oxidant, and inflammatory activities, clear evidence suggests the involvement of modified AGE proteins in degenerative disorders such as neurodegenerative pathologies, cardiovascular disease, and diabetes complications [23,24,25]. At the cellular level, AGEs can contribute to these pathologies in two different ways: (1) by binding specific receptors on cell membrane, mainly the receptor for advanced glycation end-product (RAGE), which trigger inflammatory and oxidative processes implicated in the pathogeny of several diseases processes, and (2) through an independent-receptor manner, cross-linking proteins and altering their structure, properties, and functions [18,26]. Indeed, as positively charged Lys and Arg residues are the preferential glycating sites, modifications at these residues not only affect their local microenvironment and the protein charge, but also shield them from the formation of hydrogen bonds that stabilize the protein structure. In addition, formation of covalent cross-links within different polypeptides strongly contribute to modify protein structure thus affecting its physiological function.

Moreover, glycation at lysine residues would also impair the clearance by the ubiquitin–proteasome system because ubiquitination of lysine residues, a modification that targets the protein to the proteasome for degradation, would be prevented in glycated proteins. In this respect, protein glycation, besides affecting protein structure and function, might also favor accumulation of proteins as aggregates or inclusion in tissues [27,28,29].

## 3. Amyloid Aggregation Process

The conversion of native soluble proteins into insoluble amyloid deposits has attracted considerable interest in the last decades as associated with several disorders known as “amyloid diseases”. So far, there are approximately 50 disorders, with a multitude of symptoms, associated with the misfolding of soluble, functional proteins or peptides, and their following conversion into amyloid fibrils [3,30,31]. This wide range of diseases includes neurodegenerative disorders like Alzheimer’s, Parkinson’s, and prion diseases, as well as non-neuropathic conditions such as type II diabetes [3,4,32]. With the increase of life expectancy, these disorders are no longer rare, but are rapidly becoming among the most common and debilitating medical conditions. In these diseases, the physiological alterations are associated with the formation of fibrillar aggregates, known as amyloid fibrils, which regardless of the protein involved, share a common ultrastructure [33,34]. These protein aggregates interfere with neuronal function and induce toxicity that ultimately drives cell death. Amyloid aggregates usually accumulate both in the intra- and extra-cellular space and promote a dual toxicity: loss of natural protein function mechanism (by improper folding, degradation or localization) and/or gain of toxic novel functional mechanism (toxic structures that accumulates in an incorrect location) [35,36].

The formation of amyloid structures is not a rare phenomenon and it reflects a well-defined structural form of the protein that is an alternative to the native state, a form that may in principle be adopted by many, if not all, polypeptide sequences [3,37,38]. Indeed, it is now believed that many, if not all, proteins can form amyloid fibrils in appropriate experimental conditions. This is because protein folding and protein aggregation, although being distinct processes, are in competition and the environmental conditions can instruct polypeptide chains on the conformation to adopt. Extensive studies have been performed in vitro to shed light on the structural transitions between natively folded states and amyloid-aggregation prone states. Natively folded globular proteins possess a small but significant tendency to convert into the amyloid state without crossing a major energy barrier for unfolding, by populating native-like conformations as a consequence of local unfolding, thermal fluctuations, or ligand release. The dangerous aggregation-prone states, although quite similar to the native state, seem to display altered surface charge distribution, alternative β-sheet topology, increased exposure of hydrophobic surfaces, and aggregation-prone sequences of the polypeptide chain [39,40,41]. The formation of the aggregation-prone conformation has been well characterized in vitro for different model proteins and it has been shown to be promoted by low pH, high temperature, high ionic strength, point-mutations, or organic solvents able to destabilize the native state [41,42,43,44,45,46].

**Figure 3:**
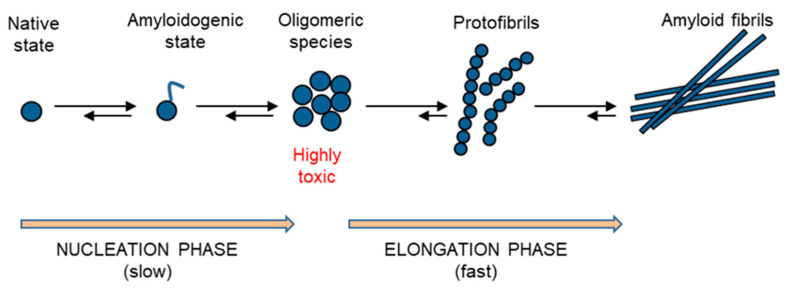
Schematic representation of the amyloid aggregation process. The process originates with the formation of amyloidogenic states, which are highly disordered and which possess an intrinsic propensity to further assembly into oligomeric species that are highly reactive (nucleation phase). The oligomeric species rapidly evolve to the formation of insoluble protofibrils and, eventually, the fibril growth proceeds by further association of protofibrils (elongation phase).

The kinetics of amyloid aggregation originates with the formation of monomeric states, highly disordered, that possess an intrinsic propensity to further assembly into oligomeric species that are heterogeneous and highly reactive. The soluble oligomeric species rapidly evolve to the formation of insoluble protofibrils and, eventually, the fibril growth proceeds by further association of protofibrils [3,32] (Figure 3). The protein fibrils, regardless of the protein sequence and structure, share common properties such as a core rich in β-sheet structure adopting a characteristic cross-β topology, the resistance to degradation and significant mechanical properties with high tensile strength. Thus, amyloid fibrils are highly resistant to in vivo degradation as extremely stable thermodynamically and thus difficult to unfold and processed by the proteasome. In addition, their thermodynamic stability contributes to convert close native proteins into amyloid species [47,48]. While insoluble amyloid fibrils correlate with disease progression, the soluble oligomeric species are generally associated with cellular toxicity as able to interact with biological membranes. The mechanisms of cellular toxicity of amyloid oligomers include both the membrane disruption, resulting in calcium imbalance, mitochondrial dysfunction and intracellular reactive oxygen species, as well as the direct interaction with membrane proteins, leading to the alteration of their native function [30,49].

Although the molecular mechanisms underlying the amyloid formation has been well characterized in vitro, poor information is available on the molecular determinants that trigger amyloid formation in vivo. Post-translational modifications such as phosphorylation, nitration, acetylation, methylation, and glycation are known to have an active role in protein aggregation as they are able to affect protein structure and function [6,7]. Clearly, the understanding of the molecular mechanisms that trigger formation and propagation of amyloid species in vivo is a prerequisite for developing new treatment options for amyloidosis.

## 4. Glycation Role in Amyloid Aggregation

Although the amyloid aggregation process has been widely studied in vitro for different amyloidogenic proteins, and many physiological (environmental and genetic) factors involved have been identified, the molecular mechanisms underlying the amyloid formation in vivo and in pathological conditions are still poorly understood. Most neurodegenerative diseases are sporadic, suggesting that external factors might contribute to the onset and the progression of these disorders. Post-translational modifications are known to affect amyloid aggregation process as able to affect protein structure and function [5,6]. Among them, protein glycation seems to have a key role in amyloid formation both in vivo and in vitro. In addition, proteins in amyloid deposits are often found glycated in patients thus suggesting a direct correlation between protein glycation and amyloidosis [9,10,11,12]. In this respect, the effect of glycation in the amyloid aggregation process of proteins related to misfolding diseases has been widely studied in order to identify the role of glycation in the in vivo amyloid aggregation and cytotoxicity. In this study, we have analyzed the most recent advances in the field for different protein models.

### 4.1. Aβ-Peptide

Alzheimer’s disease is characterized by the deposition in the brain of senile plaques, composed largely of the β-amyloid peptide (Aβ). The Aβ amyloid aggregation originates from an unstructured random coil conformation (monomeric form) that rapidly proceeds to β-sheet structure during aggregation (oligomers, protofibrils, fibrils) [50,51]. Although the Aβ-induced neurotoxicity is directly responsible for the pathology of AD, the in vivo toxic forms of Aβ-peptide remain poorly characterized. Non-enzymatic glycation seems to play a key role in the in vivo Aβ-toxicity. Indeed, the amyloid plaques in the AD brains are colocalized with AGEs and the plaque enriched fractions contain approximately threefold higher AGE adducts than that of the age-matched controls, suggesting that Aβ may be glycated in AD [12,52,53]. The long-live proteins are preferentially modified to form AGEs and the high stability of Aβ makes it an ideal substrate for non-enzymatic glycation and formation of AGEs. A role of blood sugars would also explain the link observed between the apparently unrelated diabetes and AD; diabetic patients have a 2-5-fold higher tendency to develop AD compared with nondiabetic individuals [54,55,56,57,58].

To clarify the role of glycation in amyloid aggregation process of the Aβ-peptide, several studies have been performed in vitro on different AGE-derived Aβ-peptide. In particular, glycation of lysine residues has been shown to strongly affect oligomers stability, secondary structural content, structural disorder, and propensities of inter-peptide salt bridges [59]. The glycated peptide resulted in a more rigid assembly associated with a greater beta-sheet component, suggesting that glycation results in structural modifications of key self-assembling entities making them more aggregation prone [59]. In this study, it has been also explored the effect of glycation on the stabilities of pre-formed protofibrillar Aβ-peptide species. Interestingly, glycation has been found to also induce major stabilizing effects on putative pre-formed protofibrillar structures corresponding to those found in brains of AD patients [59].

Differential effects of glycation between single-lysine and double-lysine CEL-modifications have been recently observed. Indeed, while little effect has been observed for single CEL-modifications (Lys-16, Lys-18) in amyloid aggregation rate, a stronger effect has been obtained in the double lysine CEL-modifications. In particular, CEL-modifications at both Lys-16 and Lys-18 promote a substantial decrease in free energy change, which contributes to fibril destabilization, and an increased aggregation rate. In this respect, the amyloid aggregation of the double CEL-modified Aβ-peptide produced a lower amount of amyloid fibrils compared to the unglycated peptide and a higher percentage of soluble oligomers [60]. Similar results (reduced amount of amyloid fibrils and a higher concentration of oligomers) have been observed in the amyloid aggregation of Aβ-peptide glycated in the presence of MGO [61].

With regards to the role of glycation on the toxicity of Aβ amyloid aggregates, only poor information is available. It is well established that the conformational state of this peptide is a key factor for its neurotoxicity. In particular, increasing evidence have suggested that the more toxic species for Aβ are the soluble oligomers both in vitro and in vivo [62,63,64,65,66,67,68].

So far, different toxicity effects have been observed for glycated Aβ aggregates accordingly to the glycating agent used. In particular, glycation performed with MGO has been shown to promote higher neurotoxicity of Aβ aggregates although this effect seems not to be associated with amyloid aggregates but to a specific upregulation of RAGE by Aβ-peptide [53]. In fact, RAGE is known to possess a surface binding site both for AGE species and Aβ-peptide [69,70]. Differently, glycation performed with glucose, fructose and chondroitin sulfate has been shown to stabilize the fibrillar aggregates in Aβ associated with very low toxicity [71]. Recent data performed on the double CEL-modified Aβ-peptide have shown that, although glycation stabilize the formation of soluble oligomers, lysine modification of Aβ abolish its neurotoxicity, and this seems to be associated with the key role of these lysine residues in the binding of Aβ with the cell membrane [60]. Even though further studies will be needed for a better understanding of the role of glycation in the toxicity of Aβ aggregates, this seems to be strongly dependent on the structural modification induced by the glycating agents.

### 4.2. α-Synuclein

α-Synuclein (α-Syn) misfolding and aggregation is a hallmark in several neurodegenerative diseases including Parkinson’s disease (PD) and dementia characterized by the presence of intraneuronal deposition of Lewy bodies (LB), and multiple system atrophy [72,73]. Although α-Syn amyloid aggregates are known to be the main component of the LB, which cause the loss of dopaminergic neurons in the disease, there is no consensus on what mechanisms trigger α-Syn in vivo aggregation, neuronal cell loss, and degeneration. Post-translational modifications such as glycation, sumoylation, and phosphorylation are known to be directly involved in α-Syn misfolding and aggregation [10,74,75,76]. In particular, glycation seems to have a key role in α-Syn aggregation as advanced glycation end-products (AGEs) and α-Syn are co-localized in the brain of the patients at both the early and advanced stages of PD [10,77]. In addition, accumulation of AGEs on LBs becomes more relevant to people suffering from diabetes mellitus (DM), which could explain the increased prevalence of PD in DM patients [78,79]. Glycated α-Syn also induces lipid peroxidation in vivo and results in lesions within cells [80,81]. For these reasons, glycation may play a key role in in the amyloid aggregation of α-Syn associated with Lewy body formation in PD [10,76].

Normally in neurons, the N-terminal domain of α-Syn adopts an amphipathic α-helical conformation that associates with membranes where it assembles into multimers [82,83,84]. This region is the one responsible for in vivo amyloid aggregation and, interestingly, it contains several lysine residues that can be glycated both in vitro and in vivo [85,86,87,88]. For this reason, several studies have been performed with different glycating agents in order to clarify the role of glycation in α-Syn in vivo aggregation. In particular, glycation performed in the presence of D-ribose, MGO, and GO as glycating agents has been shown to strongly stabilize the N-terminal domain thus inhibiting amyloid fibril formation in α-Syn. Despite restraining fibril formation, glycation was promoting the formation of stable cross-linked oligomeric species, able to induce oxidative stress and high cytotoxicity [85,86,89]. Most likely, the structure of this aggregates hinders further aggregation and makes the formation of amyloid fibrils difficult. The fact that glycation of α-Syn leads to the formation of stable oligomers and inhibits fibril formation can be relevant for the study of synucleinopathies as α-Syn oligomers are more toxic than larger aggregates [90,91]. Different results have been recently obtained in the CEL-modified α-Syn that is unable to form both amyloid fibrils and oligomeric species [92]. In this study, the different behavior observed upon glycation could be due to the fact that the fifteen lysine residues in α-Syn were synthetically modified with CEL moieties thus not able to form protein cross-links underlying the oligomers formation observed with D-ribose, MGO, and GO. The CEL-modifications were shown to strongly stabilize the α-Syn structure and even the presence of zinc, able to induce amyloid aggregation in α-Syn, was unable to promote aggregation in the CEL-modified protein [92].

Recently, the effect of glycation in α-Syn has been investigated in vivo using different models [77]. This study has shown that glycation might play an important and underappreciated role in PD and other synucleinopathies by modulating α-Syn biology. In particular, glycation is proven to also primarily affect the N-terminal region of α-Syn in animal models, thus reducing its ability to bind to lipid membranes. In this way, glycation promotes α-Syn accumulation and formation of oligomers that result as highly cytotoxic both in human cell lines and, importantly, in differentiated patient-derived iPSCs [77]. The toxic effects of glycation in α-Syn aggregation were also detected in animal models of Parkinson’s disease: α-Syn transgenic drosophila and mice. Indeed, in α-Syn expressing flies, glycation reduces both the motor performance and survival while in mice, MGO injection in the substantia nigra causes an impressive loss of neuronal cells. Besides stimulating the accumulation of toxic α-Syn oligomers that impair neuronal synaptic transmission, glycation is proven to block α-Syn ubiquitination thus also impairing its clearance and release [77]. Taken together, these findings confirm that glycation may perturb the physiological role of α-Syn on vesicular trafficking by promoting protein accumulation and amyloid aggregation associated with LB.

### 4.3. Insulin

Insulin amyloid-like fibrils are the hallmark of a clinical condition observed in insulin-dependent diabetic patients, called insulin injection amyloidosis in which insulin fibrils are found at the site of insulin injections [93,94,95]. Native insulin is mainly organized in an α-helical structure and its amyloid aggregation is proposed to occur via partial unfolding of a monomeric intermediate that promotes protein oligomerization and the α to β transition underlying the amyloid formation [96,97,98].

Insulin is associated with glycemia and is susceptible to in vivo glycation by glucose and other highly reactive carbonyls especially in diabetic conditions [99,100,101]. When glycated, human insulin is unable to regulate glucose homeostasis and stimulate glucose transport and adipose tissue lipogenesis [102,103,104]. Indeed, glycation has been reported to affect insulin structure, stability and amyloid aggregation depending on glycating agent and/ or environmental conditions. Insulin can be glycated by glucose in vitro and glycated species possess different structural features depending on the experimental conditions used [105,106,107]. In particular, glycation in reducing conditions promotes insulin oligomerization thus accelerating amyloid aggregation, while, in non-reducing conditions, it strongly inhibits amyloid formation in a way proportional to the glycation extent [107]. Human insulin can be also glycated by MGO that promotes the formation of native-like species and reduces the ability of insulin to form amyloid fibrils by impairing the formation of the seeding nuclei. Although MGO reacts with a single residue in insulin (Arg22), it strongly stabilizes the native structure as glycated species are soluble, non-fibrillar and retain a native-like structure [108]. A similar effect has been observed when glycation is performed in the presence of D-ribose, able to react with N-terminus and Lys29 in human insulin. Indeed, glycation by D-ribose strongly stabilize insulin native structure and impair the α to β transition underlying the amyloid formation [109]. The overall data suggest that, at least in non-reducing conditions, glycation seems to have a protective effect in insulin amyloid formation as it is able to stabilize insulin-native structures, thus preventing amyloid aggregation. Moreover, as accumulation of AGEs has been suggested as one of the main responsible factors of diabetes-associated complications, such as retinopathy, nephropathy, and atherosclerosis, further examination of the molecular bases underlying the toxic effect produced by AGE-modified insulin on neighboring cells might help to identify new therapeutic interventions.

### 4.4. Islet Amyloid Polypeptide

The presence of amyloid fibrils in pancreatic β-cells, arising from the aggregation of human islet amyloid polypeptide (hIAPP), is a hallmark of type 2 diabetes (T2DM) [110,111]. hIAPP is a 37-residue natively unstructured polypeptide that is prone to aggregate into amyloid fibrils, thus inducing pancreatic β-cell dysfunction, cell death, and loss of islet β-cell mass. Therefore, the aggregation of hIAPP is considered one of the major causes of T2DM [112,113,114,115,116,117]. The amyloidogenicity of IAPP is very sensitive to residue changes or post-translational modifications including glycation [118,119,120,121,122,123].

hIAPP is likely to be glycated in vivo as AGE immunoreactivity colocalizes with regions of immunoreactive IAPP-derived amyloid and it has been proposed that AGE-modified IAPP acts as a template in the nucleation-dependent aggregation of IAPP into amyloid fibrils. In particular, in vitro glycation of IAPP with glucose has been shown to promote the formation of protein aggregates showing a better seeding efficiency than freshly dissolved IAPP and also exhibited higher cytotoxicity than control IAPP [118,119,120]. Similar data were recently obtained on the chemically synthesized AGE-IAPP by modification of Lys1, the only Lys residue, with carboxymethyl-lysine (CML) AGE [124]. Indeed, this CML-modified IAPP was forming amyloid aggregates faster than non-modified IAPP, and higher molecular weight AGE-IAPP oligomers were also observed in the early stage of aggregation. In addition, AGE-IAPP can promote amyloid aggregation in non-modified IAPP, and its fibrils can also act as templates to trigger IAPP aggregation. Moreover, the AGE-modified IAPP, such as normal IAPP, is able to interact with synthetic membranes and also to exhibit cytotoxicity [124]. Recently, glycation of IAPP has been studied in the presence of MGO as glycating agent [125]. In this study, MGO has been shown to efficiently react only with IAPP Lys1 inducing both a slowdown of the IAPP aggregation process and changes in the aggregate morphology [125]. This study suggests that, although the only AGE-modified residue is Lys1 as in the CML-derived IAPP, differences in the AGEs produced and in the experimental conditions may play a key role in the dynamic effects induced by glycation on the aggregation process. The overall data suggest that glycation modifications of hIAPP might strongly modulate the amyloidogenic properties of this protein, and this could play a key role in accumulating additional amyloid during T2DM progression.

### 4.5. Albumin

Human serum albumin (HSA), the most abundant serum protein with versatile applications both in vivo and in vitro, has been a widely used model for understanding the structural effects of glycation as it contains 83 potential glycation sites (59 lysine and 23 arginine residues, N terminus) [126,127,128,129]. Glycated HSA accounts for 80% of the circulating glycated protein and it has been implicated in several complications associated with diabetes [130,131,132]. Although HSA is a highly soluble protein mainly organized in α-helical structure, it is able to form amyloid fibrils through partial unfolding of the tertiary structure and conformational changes of the secondary structure [133,134,135]. In addition, this protein can be efficiently glycated in vitro by several glycating agents as glucose, D-ribose, MGO and GO and similar effects on the amyloid propensity have been observed with all of them [126,127,128,136,137,138]. In particular, glycation has been shown to promote strong conformational changes in HSA that affect both secondary and tertiary structure and markedly reduce the protein stability. In this way, glycation promotes amyloid aggregation in HSA both reducing the helical content and supporting the formation of β-cross structure that rapidly evolve to the formation of amyloid aggregates [129,139,140,141]. Interestingly, glycation of albumin performed in the presence of D-ribose, besides promoting amyloid formation, has been shown to stabilize the amyloid oligomeric species that result highly cytotoxic in neuronal cells [129]. Indeed, amyloid aggregates of ribosylated albumin were able to induce oxidative stress ROS-mediated and apoptosis in neurotypic cells. The overall data suggest that non-enzymatic glycation reaction could have a key role in the in vivo BSA glycation as both promoting amyloid formation and stabilizing toxic oligomeric species.

### 4.6. Superoxide Dismutase 1

Amyloid aggregation of copper, zinc superoxide dismutase SOD1, an essential component of the cellular antioxidant defense system, is associated with amyotrophic lateral sclerosis (ALS), a neurological disease causing the death of motor neurons and muscular paralysis. ALS it is predominantly a sporadic disease, in some cases (10%) it has been described as familial [142,143,144].

In vitro studies have shown that human SOD1, when lacking both its metal ions (ApoSOD), forms amyloid aggregates under physiological conditions of pH and temperature. Indeed, loss of metal binding not only induces protein unfolding and loss of enzymatic activity in SOD1, but also promotes amyloid formation [145,146,147,148,149]. Although the molecular mechanisms underlying amyloid aggregation of SOD1 has been widely studied in vitro, poor information is available on the mechanism that trigger amyloid formation in vivo and in the pathological conditions of ALS.

In this respect, SOD1 glycation seems to have a determining role both in sporadic and familial forms of ALS; in fact, spinal cord and brain samples have been found to be glycated in patients [150,151]. Moreover, SOD1 has been shown to be glycated in vivo and glycation sites have been identified and they are six lysine residues (number 3, 9, 30, 36, 122, 128) spread along the protein sequence [152]. SOD1 glycation has been studied in vitro in the presence of different glycating agents and the effects of glycation in amyloid aggregation has been also evaluated [153,154,155]. In particular, SOD1 can be efficiently glycated in vitro by glucose, D-ribose, GO, and MGO and glycation was shown to promote protein unfolding, loss of copper binding and inhibition of the enzymatic activity [154,156,157,158]. These results have been confirmed by ex vivo experiments reporting that SOD1 extracted from erythrocytes of diabetic patients was significantly more glycated and has a lower enzymatic activity, with respect to controls [159]. Although promoting SOD1 unfolding and loss of metal binding, glycation has been shown to inhibit amyloid aggregation in SOD1 and promote the formation of stable cross-linked AGEs [153,155]. Taken together, these results suggest that glycation, besides having protective role in SOD1 amyloid aggregation, could impair the correct maturation of SOD1 in vivo as promoting protein unfolding, demetallation, and loss of enzymatic activity, thus triggering cellular oxidative stress.

### 4.7. Lysozyme

Amyloid aggregation of human lysozyme is responsible for lysozyme amyloidosis, a non-neuropathic hereditary amyloidosis in which protein mutations favor the formation of misfolded conformers which in turn leads to lysozyme aggregation and accumulation of amyloid deposits in several organs [160,161,162,163,164]. Hen egg white lysozyme (HEWL), a structural homolog of human lysozyme, has been widely used to study the amyloid aggregation of lysozyme in vitro. Wild-type HEWL is a globular protein with antibacterial activity and has a low aggregation tendency under physiological-like conditions, although it can form amyloid fibrils in denaturing conditions able to destabilize the native structure of the protein [165,166,167,168]. At the same time, this protein possesses six Lys as potential glycation sites and, for this reason, HEWL represents an ideal model to study if glycation per se induces amyloid aggregation.

HEWL has been shown to be susceptible to glycation by several glycation agents, such as D-glucose, D-ribose, D-fructose, and MGO [169,170,171]. Glycation strongly affects the structure of HEWL and inhibits its enzymatic activity thus increasing the susceptibility to bacterial infections [172,173]. Moreover, glycation affects the amyloid aggregation process of HEWL. Generally, glycation promotes the formation of oligomeric aggregates in HEWL but the mechanism underlying the process depends on the chemical nature of the glycating agent due to the different residues involved in the AGEs formation.

At first, the effect of glycation on HEWL aggregation has been studied using D-glucose, D-fructose and D-ribose as glycating agents and glycation was found to promote the formation of oligomeric species stabilized by covalent cross-links [174,175]. In these studies, it was hypothesized that glycation was inducing partial unfolding in HEWL promoting the α- to β-transition underlying oligomeric formation. However, through a more detailed study, it has been shown that HEWL ribosylation involves a chemical multistep conversion that induces covalent modifications on lysine side chains without altering the protein structure but changing the protein charge and enlarging its hydrophobic surface. The increase of surface hydrophobicity triggers the assembly of ribosylated HEWL into native-like small spherical oligomers highly toxic, which further evolve into insoluble native-like protofibrils [176].

The glycation of HEWL has been also performed with glycolaldehyde as glycating agent and two different effects on protein aggregation were observed depending on HEWL concentration regime [177]. In particular, at low HEWL concentration (below 2μM), non-cross-linking fluorescent AGEs were formed on Lys side chains, and they did not affect the protein structure but inhibit its enzymatic activity. These AGEs were having little impact on HEWL surface hydrophobicity and, therefore, a negligible effect on its aggregation propensity. Upon increasing HEWL concentration (20–100 μM), the glycation mechanism was shifting toward the formation of intermolecular cross-links, which trigger a polymerization cascade involving the formation of insoluble spherical-like aggregates through a concentration-dependent nucleation mechanism [177]. Recently, a different effect on glycation mediated HEWL aggregation has been reported using MGO as glycating agent [178]. In this study it has been shown that, upon incubation with MGO for three weeks, the stress-induced aggregation of HEWL was strongly reduced. Indeed, upon thermal and chemical stress, while the non-modified HEWL was rapidly forming amyloid fibrils, the MGO-modified protein was not showing any fibril formation but only small amorphous structures. Structural analysis has shown that MGO-glycation only affects arginine residues and induces changes in tertiary structure of the protein without significantly affecting its secondary structure. The authors have hypothesized that the MGO-induced modification of HEWL arginine residues to neutral AGE adducts could be responsible for the reduced susceptibility to amyloid aggregation [178]. These results notably differ with the aggregation-modulation mechanism of ribosylated HEWL directed by hydrophobic interactions clearly showing that the mechanism underlying the aggregation of a glycated protein strongly depends on the chemical nature of the glycating agent.

### 4.8. Hemoglobin

Human hemoglobin (Hb) has been the first glycated protein to be identified in vivo and it is widely used in diagnostics as it indicates the presence of excessive sugar in the bloodstream [179,180]. Indeed, the subfraction HbA1c, glycated at the amino-terminal valine residue of the β chain, may be significant with increased level of blood glucose over prolonged periods of time and, for this reason, is considered an important biomolecular marker for evaluating long term control of diabetes.

Hb can be glycated by glucose, fructose, and methylglyoxal [181,182,183], and several studies have been published on glycation-induced structural and functional modifications of Hb [182,184,185]. In particular, it has been shown that the glucose-induced glycation (both in vitro and in vivo) promotes iron release and enhances free-radical mediated oxidative stress [181]. Exposure to fructose directly promotes strong conformational changes in Hb driving the native α-helical structure into non-native often β-sheet rich structure, favors the unfolded conformation and stimulates Hb aggregation [182,186,187]. Glyoxal is the more efficient glycating agents so far tested for Hb and it has been shown to promote considerable retention of secondary structure and loss of tertiary structure as suggested by increased heme exposure and reduced hydrophobic surface. Glycation by glyoxal also promotes the formation of partially unfolded molecules that are aggregation-prone thus inducing aggregation in Hb [188,189]. The overall data suggest that glycation modifications of Hb might strongly modulate the amyloidogenic properties of this protein, and this could play a key role in accumulating additional amyloid during diabetes progression.

## 5. Conclusions and Perspectives

Protein glycation is a spontaneous age-dependent post-translational modification that can affect the structure and function of several proteins. Glycation reaction is strongly affected by concentration and reactivity of the glycating agent (reducing sugar, carbonyl compound), oxidative conditions, number of glycation sites, and their exposure in the protein structure. In this respect, the effect of glycation on the protein structure strongly depends on the exposure of glycating sites in the protein structure, the type of AGEs formed, and the environmental conditions. The data presented for the different model proteins clearly suggest that glycation affects the aggregation properties of polypeptides unevenly; it stimulates amyloid aggregation in some proteins, whereas it slows down the process for other proteins. Indeed, glycation is shown to accelerate the aggregation rate in some model proteins as Aβ-peptide in the double lysine CEL-AGE, IAPP in the presence of glucose and hemoglobin in the presence of fructose and GO [60,120,182,186]. At the same time, glycation can inhibit amyloid aggregation as in IAPP with MGO; in α-synuclein with D-ribose, MGO, and GO; in insulin with D-ribose and MGO; and in SOD1 with glucose, D-ribose, GO, and MGO [85,86,107,108,156,158,182,186]. In addition, glycation has been also shown to promote amyloid aggregation in natively folded proteins as in human albumin in the presence of glucose, D-ribose, MGO, and GO [126,127,128,136,138]. The different effect can be ascribed both to the type of AGE formed and, also, to the molecular mechanisms underlying the aggregation process of the protein involved. Indeed, being a post-translational modification, glycation to specific residues can affect amyloid aggregation and the related cytotoxicity if the residues or their microenvironment are directly involved in the amyloid formation. In addition, protein glycation seems to strongly stabilize protein aggregates make them more difficult to remove from the proteasome system.

As oxidative stress is a triggering factor both in the glycation reaction and in the amyloid formation and induced toxicity, a potential therapeutic strategy for amyloid diseases could involve the use of molecules with antioxidant activity. In this respect, much attention has been paid to natural compounds, such as polyphenols, well known for their antioxidants and anti-inflammatory properties, able to prevent/protect by neurodegenerative diseases [190,191,192,193,194,195,196]. Several studies have suggested that natural phenolic compounds can interfere both with protein glycation and with the amyloid aggregation process of several model proteins [194,197,198,199,200,201,202,203,204,205,206]. In this context, polyphenols could represent an efficient additional therapy capable of acting on several mechanisms common to the pathologies of diabetes and amyloidosis simultaneously. They also have the advantage of being naturally found in the diet, so that their therapeutic implementation can be through a dietary alteration or nutritional supplements, which is more cost-effective, easier to implement, socially acceptable, and generally safer.
